# Supplementary material for: How patients interpret early signs of foot problems and reasons for delays in care: Findings from interviews with patients who have undergone toe amputations
Source: PLoS One. 2021 Mar 10;16(3):e0248310. doi: 10.1371/journal.pone.0248310 (PMC7946282; doi:10.1371/journal.pone.0248310)
Supplement: S1 File — (DOCX) [file pone.0248310.s001.docx]

**S1 File.** INTERVIEW GUIDE

*Generic prompts: If responses are overly brief or require clarification, use probes to elicit more detailed responses. Probes should use words or phrases presented by the participant using one of the following formats:*

- *What do you mean by ____________ ?*
- *Can you tell me more about ____________ ?*
- *Can you give me an example of ____________ ?*
- *Can you tell me about a time when ____________ ?]*

*What happened next?*

**According to our records, you had a toe amputation on [date]. It that right? Tell me about it.**

**Probe:**

- What led up to the amputation?

If needed:

- Walk me through from the beginning.
- What, if anything, did you notice?
- What did you do?
- Where did you go?
- Who did you see?
- What happened next?
- What do you think caused your toe amputation?
- What (if anything) could have prevented your toe amputation?
- How long were you in the hospital?
- What happened next (discharge?)

*IF NEEDED:*

Looking back, is there anything you wish you had done differently? If so, tell us about that.

Looking back, is there anything you wish your health care providers had done differently? If so, tell us about that.

Is there anything else that you think we should know about your experience with your toe amputation or your recovery?
