# Supplementary material for: How patients interpret early signs of foot problems and reasons for delays in care: Findings from interviews with patients who have undergone toe amputations
Source: PLoS One. 2021 Mar 10;16(3):e0248310. doi: 10.1371/journal.pone.0248310 (PMC7946282; doi:10.1371/journal.pone.0248310)
Supplement: Attachment — Submitted filename: Response to reviewers.docx [file pone.0248310.s002.docx]

Response to Reviewers

1. Please ensure that your manuscript meets PLOS ONE's style requirements, including those for file naming. The PLOS ONE style templates can be found at

https://journals.plos.org/plosone/s/file?id=wjVg/PLOSOne_formatting_sample_main_body.pdf and

<https://journals.plos.org/plosone/s/file?id=ba62/PLOSOne_formatting_sample_title_authors_affiliations.pdf>

**** Response: We have re-reviewed the style requirements and done our best to abide by them.**

2. In the Methods section, please provide a copy of the interview guides as Supporting Information.

**** Response: We inadvertently failed to include the Supplemental File with the first submission. We apologize. The interview guide is included as S1 File.**

Furthermore, When reporting the results of qualitative research, we suggest consulting the COREQ guidelines: http://intqhc.oxfordjournals.org/content/19/6/349. In this case, please provide additional information on how participants were approached for the interview, and where the interviews took place.

**** Response: We appreciate the suggestion. We consulted the COREQ guidelines to ensure that we included the key information and presented it as clearly as possible; we made changes where we noticed opportunities to clarify our methods. We added a new subheading “Recruitment” where we detail how patients were approached. We state on line 127 that interviews were conducted over the telephone.**

3. Please provide additional details regarding participant consent. In the ethics statement in the Methods and online submission information, please ensure that you have specified what type you obtained (for instance, written or verbal, and if verbal, how it was documented and witnessed). If your study included minors, state whether you obtained consent from parents or guardians. If the need for consent was waived by the ethics committee, please include this information.

**** Response:** **We now specify that we obtained verbal consent and explain that we obtained a waiver of documentation of written informed consent in lines 114-115. Consent was verified at the start of the interview and recorded.**

4. We note that you have indicated that data from this study are available upon request. PLOS only allows data to be available upon request if there are legal or ethical restrictions on sharing data publicly. For information on unacceptable data access restrictions, please see http://journals.plos.org/plosone/s/data-availability#loc-unacceptable-data-access-restrictions.

**** Response: Due to legal and ethical restrictions, we are unable to share data publicly because the data contain potentially identifying and/or sensitive patient information.  Data access requests will be reviewed by the IRB of the VA Puget Sound Health Care System (contact via Dr. Littman).**

Reviewer #1: Interesting qualitative study that was well designed.

Generalizability is always an issue for these types of studies. As is common for this type of research a large number of potential subjects were not ultimately participants. Only men were interviewed. Need to be careful about the implication of using the term “large national sample”. While it is, this term usually implies that the sample is representative, and this is rarely true for a qualitative study including this one.
**** Response: The reviewer makes a good point that our sample may not be representative. In line 405, we now state, “In interviews with a large sample of male patients from across the United States with diabetes who had undergone a toe amputation, delays in care were common.” We also include generalizability as a potential limitation in lines 444-445.**

It is interesting that patient understanding their medical condition was an issue for patients who are receiving education. This has been a common issue for educational programs and likely means we need to do a better job by maybe doing a “different” job.

**** Response: We agree that it is interesting that patients understanding their medical condition was an issue for patients who (we believe) are receiving education. As noted by the reviewer, this has been a common issue for education programs and suggests that more research is needed to determine how to make educational programs more effective.**

Reviewer #2: Very well presented! It is rare that the authors have paid attention to the details of manuscript preparation. Thank you for your work and growing the edge of knowledge with your work. I look forward to seeing this in print!
Just an edit:
Pg5 line 115 do you mean "inquiry" rather than "analysis"
A pleasure to read!

**** Response: We are pleased that you appreciated our paper. In prior line 115 (current line 130), we have replaced the word “analysis” with “inquiry.”**
